# Analysis of the benefits and production challenges of working donkeys in smallholder farming systems in Kenya

**DOI:** 10.14202/vetworld.2020.2346-2352

**Published:** 2020-11-06

**Authors:** Mary Gichure, Joshua Onono, Raphael Wahome, Peter Gathura

**Affiliations:** 1 Department of Animal Sciences, Chuka University, P.O Box 109-60400 Chuka, Kenya; 2 Department of Public Health, Pharmacology and Toxicology, University of Nairobi, P.O. BOX 29053-00625, Kangemi, Kenya; 3 Department of Animal Production, University of Nairobi, P.O. BOX 29053-00625, Kangemi, Kenya

**Keywords:** benefits and challenges, income, livelihoods, working donkeys

## Abstract

**Aim:**

The aim of the study was to determine the benefits of keeping donkeys and associated production challenges under a smallholder farming system in Kenya.

**Materials and Methods:**

A descriptive study was conducted with smallholder farmers keeping donkeys in 13 administrative locations in Kirinyaga County. Data were collected using a questionnaire guide in 13 focus group discussions (FGDs) using participatory epidemiological methods. The FGDs comprised 8-12 participants who were donkey owners. Data were collected through listing, pair-wise ranking, and probing on the benefits of keeping donkeys, challenges faced by working donkeys and the common diseases that affect donkeys in these farms. Data analysis was performed using Kruskal–Wallis non-parametric method to test whether median ranks were significantly different. Other farm level data were also collected using the structured questionnaire and these were analyzed using descriptive statistical methods.

**Results:**

The identified benefits included income obtained from the use of donkeys in transportation (Z=5.80) and manure production (Z=3.47), which enabled the farmers to participate in trade activities and improve crop farming. The identified challenges included theft for slaughter (Z=5.99), disease incidence (Z=3.03), road accidents (Z=2.83), and malicious cutting (Z=2.32). Some of the diseases identified were tetanus (Z=5.35), hoof problems (Z=4.55), helminthiases (Z=3.10), and mange (Z=2.24). Participants ranked diseases based on their effects on work output for the donkeys, reducing productivity and often causing death. Addressing these production challenges would optimize donkey use among smallholder farmers.

**Conclusion:**

The results presented can be important for policymakers and extension agents regarding the health and welfare of donkeys kept under similar settings.

## Introduction

The estimated world population of working donkeys is 44 million, with 13.7 million found in Africa [1] and 1.8 million in Kenya [2]. A majority of working donkeys are owned by individuals as a source of income, which makes significant contributions to individual households and national economies [3]. The disposable income enables many families to access basic needs for survival such as food, clothing, and shelter [4]. Working donkeys also support other households’ income generation activities, particularly livestock and dairy production [3]. Humans benefit directly from livestock as a source of food such as milk and meat and indirectly through income generated from the sale of animals and their products. For working animals, draught power is an important output although it is not included among the primary outputs/products of working equines [5]. There is an increasing demand for donkey skin in Asian countries for the production of traditional medication known as *ejiao* [6]. Donkeys are considered as food animals [7] and their meat was legalized in Kenya in 1999 [8]. Their meat has not been accepted for consumption in many parts of Kenya but it is an accepted delicacy among the Turkana community in Kenya [9]. The consumption of donkey meat is popular in China and the meat is highly-priced [10]. The donkey meat is low in fat and cholesterol and rich in iron [11]. In addition, milk from donkeys is used among the Maasai community in Kenya where it is fed immediately after milking, while still warm to children to manage severe cough or pneumonia or to prevent diseases such as the common cold among them [12]. Donkey milk was reportedly fed to orphaned children in Paris in the 19^th^ century [13]. Donkey milk is reported to aid in the inactivation of certain viruses, bacteria, and tumors due to the lysozyme enzymes present in it [14]. Working donkeys are faced with challenges such as poor husbandry and management, improper, and often injurious working implements including improper harnesses and cart design [15]. Donkeys are also mistreated through whipping, overworking, overloading, straying, poor feeding, and poor handling practices in the form of the use of tether ropes [16]. Reports have also been published where donkeys suffer diseases such as trypanosomiasis [17,18], parasitic infections [19-22], and African Horse sickness [23]. However, in Kenya, another emerging challenge is the theft and inhumane slaughter of donkeys by unscrupulous traders who are part of a larger international network of trade in donkey skin and meat [24,25], which if not properly checked will threaten the donkey population within the continent of Africa, and globally [4]. Furthermore, these illicit activities of trade also present welfare challenges for donkeys.

It needs to be recognized that the benefits of keeping donkeys and the associated production challenges inherent in these production systems may vary across production systems. Donkeys are raised in different agro-ecological systems in Kenya ranging from arid and semi-arid areas and highland agro-ecological areas. These donkeys are used for work within rural, peri-urban, and urban areas, where they complement transport services. The current published literature has generalized the benefits and associated challenges of working donkeys, but may omit significant benefits and production challenges in other production areas.

The study was conducted with the aim of describing the types of benefits and the associated production challenges of working donkeys perceived by owners, within the context of smallholder farming systems within the peri-urban and rural areas in the central highlands agro-ecosystems of Kenya. The results from this study are useful for veterinary practitioners and policymakers to support donkey health and welfare in prioritizing the benefits and production challenges of keeping donkeys for livelihoods who depend on donkeys for sustenance.

## Materials and Methods

### Ethical approval and informed consent

Ethical approval to conduct the study was granted by the University of Nairobi, Faculty of Veterinary Medicine, Biosafety Animal Use and Ethics Committee (FVM BAUEC/2018/165). In addition, before conducting the discussions, the study objectives were introduced to the participants and verbal consent to participate in the discussions was granted from the participants. Permission to conduct the discussions in the villages was also obtained from the village chiefs before the commencement of the study.

### Research design and study area

This descriptive study was conducted during the months of June to September 2018 in Kirinyaga County. The County borders Mt Kenya and is divided into three ecological zones. The lowland areas (1158-2000 m above sea level [ASL]) that are characterized by gentle rolling plains, the midland area (2000-3400 m ASL) and the highland area (3400-5380 m ASL), which include the whole of the mountainous area. Due to challenges with the topography of the area, donkeys are found within the lowlands and some parts of the midlands, where they were used as a means of transport by smallholder farming households. The county has a human population of 528,054, occupying an area of 1,205.4 km^2^, with a donkey population 3,990 [2]. Administratively, the county is divided into five subcounties, which are further subdivided into 12 wards, 30 locations, and 81 sublocations. Out of the thirty locations in Kirinyaga County, 13 locations were purposively selected because of the presence of large numbers of donkeys raised there.

### Selection of study units

Preliminary visits to the selected study locations were conducted with local authorities (chiefs) and leaders of donkey owner community groups to introduce the project and its objectives. During the visits, the chiefs and donkey owners were asked to nominate one person per village who would participate in the focus group discussions (FGDs). This selection of participants was made to identify people who could provide reliable data on the types of benefits and challenges facing donkey keepers. These participants were selected to represent the entire location. The participants consisted of donkey owners who were also donkey users. In addition, they had to be 18-years old and above and residents in the village. The researcher was not involved with the selection of the study participants.

### Data collection

Data were collected in 13 focused group discussions in the 13 selected locations. One FGD was conducted per location comprising 8-12 participants from different villages. The group discussions were guided by a checklist of open-ended questions. The responses were also open-ended and further probing was done to provide detailed data on the topics being discussed as well as to ensure a clearer understanding of the data obtained. The responses were ranked using simple ranking and pair-wise ranking methods based on the order of importance according to the participants. Key questions addressed included the benefits of keeping donkeys, the list of diseases affecting donkeys, and the challenges facing the donkeys in the study area. Additional questions asked included the types of transported materials, the reasons for ranking of the diseases that were obtained, and the proposed solutions for the identified production challenges. Data were collected by taking manual notes on flip charts based on the responses provided.

### Statistical analysis

Notes from the FGD were first transcribed into separate templates created in Microsoft Word and Excel spreadsheet (version 2010) (Microsoft Corporation, USA). The scores and ranks were then converted to reciprocals to give weight to the obtained scores and ranks. The data were then exported to Genstat statistical package for analysis (https://www.genstat.co.uk). The analysis was accomplished using the Kruskal–Wallis one-way analysis of variance to test whether the median ranks for the various benefits and challenges were significantly greater than the median score. The responses were considered significant when the computed Z-score was greater than the critical value of Z〈=1.96. Additional responses to the open-ended questions were presented in narrative summaries to support the ranks and scores obtained.

## Results

### Determination of the benefits of keeping donkeys

Donkeys kept in Kirinyaga County were used mainly as a means of transportation (Z=5.80) either for domestic transport or commercial transport which was a source of income. Donkeys were also kept by households for manure production (Z=3.47). This manure from donkeys was often used as fertilizer for farmed crops such as rice, which is produced by most families within the low laying areas of the county. Other uses of donkeys by owners and users in the area are shown in Table-1.

**Table-1:** Benefits of keeping donkeys according to smallholder farmers in Kirinyaga County from June to September 2018.

Benefits of keeping donkeys	Median rank	Z score
Transport	123.0	5.80*
As a source of manure	99.9	3.47*
For breeding purpose	72.6	0.71
For ploughing	64.9	-0.07
For sale	51.2	-1.44
Trading	51.2	-1.44
As a source of income/ to hire it out	50.1	-1.56
As a family asset	48.8	-1.68
As an identity	48.3	-1.73
As a pet	45.2	-2.05

* Significant benefits. Rice was the most frequentlytransported farm produce (10/13 groups). The rice was transported at different stages such as rice seedlings between different farms, paddy rice from the farms to the millers, and milled rice from the millers to local retailers. Water was also frequently transported (9/13) to the households. Other items transported included building materials, manure from farms, farm produce such as maize, vegetables and potatoes, as well as moving people (especially household items, sick people, and during occasions such as political campaigns and wedding ceremonies) and other animals

Donkeys were also kept for production of manure (12/13 groups), for breeding (7/13) to obtain replacement stock; as family assets (6/13) to sell it at times of money need; and for ploughing (6/13) where they substituted and complemented bulls. One group also indicated that they used donkey milk, which was thought to possess medicinal properties for people who had respiratory tract health infections.

Donkeys, therefore, contribute as a source of income to the households either through charging for the transport services they offer, or through their sale or sale of their products. Domestically, donkeys were used to avoid transport charges of fees that would be incurred by a household if farm labor was hired and hence this acted as savings for the household who used their own donkeys.

### Determination of challenges experienced by working donkeys

The challenges facing working donkeys in Kirinyaga County were theft and slaughter (Z=5.99), diseases (Z=3.03), road accidents (Z=2.83), and malicious cutting (Z=2.32) as indicated in Table-2. They are sorted in descending order of significance.

**Table-2:** Challenges experience by working donkeys according to smallholder farmers in Kirinyaga County from June to September, 2018.

Challenge	Median rank	Z score
Theft and slaughter	214.0	5.99*
Diseases	163.2	3.03*
Road accidents	159.7	2.83*
Malicious cutting	150.9	2.32*
Competition by tuk-tuk	141.5	1.77
Lack of reliable vet services	110.7	-0.02
Poor image of donkeys	101.1	-0.57
Conflicts eg donkey detentions	93.6	-1.01
Lack of feeds	92.8	-1.06
Cost and availability of treatment	92.2	-1.09
Poor payment by customers ie debts	92.1	-1.10
Lack of housing	84.6	-1.54
Harassment by police	81.3	-1.73
Theft only	81.2	-1.73
Lack of unity among peers	76.5	-2.01
Poor roads	76.5	-2.01
Seasonality of work/ weather	75.4	-2.07

* Significant challenges

Most of the respondents linked the challenge “donkey theft and slaughter” to the opening of slaughterhouses in Nakuru and Baringo Counties of Kenya and the export of donkey skin. Due to the threat of reduction of the number of donkeys raised in the country and the upcoming industrialization, most donkey owners have diversified to tuk-tuks (tuk-tuks are motorized tricycles used for transporting people and farm items) and motorbikes for transport due to the changing customer needs for increased speed and transport of lighter loads.

### Disease conditions affecting donkeys

Donkeys raised in Kirinyaga County faced diseases such as tetanus (Z=5.35), hoof problems (Z=4.55), helminthiasis (Z=3.10), and mange (Z=2.24); among other diseases indicated in Table-3.

**Table-3:** Identified disease conditions affecting donkeys according to smallholder farmers in Kirinyaga County from June to September 2018.

Disease/ Condition	Mean rank	Z score
Tetanus	191.0	5.35*
Hoof problems	178.0	4.55*
Worms	154.6	3.10*
Mange	140.7	2.24*
Wounds	120.3	0.98
Rabies	113.4	0.55
Colic	100.1	-0.27
Respiratory problems	99.7	-0.30
Diarrhea	81.1	-1.45
Eye problems	79.1	-1.57
Trypanosomiasis	75.2	-1.81
Sarcoids	71.7	-2.03
Staggering/ gaits	67.4	-2.30
Abscess, Blisters	66.7	-2.34
Allergies	66.3	-2.36

* Significant diseases

Most donkey owners in Kirinyaga County were organized in self-help groups, and they had obtained some form of training on early disease reporting and home-based care by a local animal welfare non-governmental organization working in Kirinyaga County for over 20 years. Those farmers who did not know the diseases were probably new owners who had acquired donkeys and had not been trained. The diseases which were identified as significant were those that were likely to cause death of the donkeys including tetanus, rabies, colic, and wounds; those diseases that affected work output and, therefore, reduced income to households, including hoof problems, worms, and respiratory problems; those diseases which were expensive to treat and manage such as tetanus, worms, mange, and wounds; and those that were zoonotic and contagious such as rabies as well as those affecting the appearance of the donkeys by affecting the skin coat, hence, reduces the price of a donkey when taken to the markets for sale. These diseases also caused the separation of donkeys by owners and discouraged the potential clients from hiring donkeys such as wounds and mange infestation.

## Discussion

Donkeys were used for transporting water, rice, and building materials among other items. Water was transported to homes, schools, hotels, and construction sites. Transport of rice aided rice farmers to reach markets and obtain a higher return from their rice which would be traded, and hence enabled these farmers to participate in trade activities; a finding which concurred with Valette [3] as well as Fernando and Starkey [8]. The rice was transported as seedlings, paddy, milled, and husks from the farms to the millers and consumers, while building materials were transported due to the growth of other towns within Kirinyaga County.

Donkey manure was also used in Kirinyaga for sale and use in the farms. The manure was reported to improve the soil quality by reducing the occurrence of crop parasites in the soil and reducing the acidity levels in rice fields. Karanja *et al*. [26] had previously reported that donkey manure significantly improved the composting process and the quality of the resultant compost for use as manure in crop fields. Manure yield could be increased through accumulation enabled by enclosing or housing of donkeys to increase the concentration of dung [27]. Some respondents in Kirinyaga County also consumed donkey milk, which was thought to be a remedy for non-specific respiratory health problems [12], although they did not have documented evidence about the medicinal qualities of the milk [14]. Donkey meat was neither accepted nor consumed in the area. This confirmed a report by Rono *et al*. [9], who recorded that most communities in Kenya did not consume donkey meat except for the Turkana community who were known to consume donkey meat. Unpublished reports indicated that donkey meat was sold fraudulently to consumers as beef by unscrupulous traders, who often had stolen donkeys, slaughtered them inhumanely under unhygienic conditions, which for the most part was meant to obtain donkey skin [4]. It was common to find donkey carcasses which had been deboned and the skin taken away [28]. This observation was linked to the opening of four donkey abattoirs, which had created a high demand for donkey skin for export to China to supply the ingredients for the preparation of *ejiao*, which is a product used by the Chinese people in traditional medicine, but no commercial value for skin and donkey meat has been reported in Africa [24]. The report further noted that with the decreasing donkey population and unintentional breeding challenges, unscrupulous businessmen turned to stealing donkeys which was reported in other parts of Kenya. The stolen donkeys were traced by the anti-stock theft unit to the donkey slaughterhouses which had been recently commissioned in Kenya [24].

Donkey theft and uncontrolled slaughter of donkeys would significantly reduce the population of donkeys in Kenya and consequently affect the livelihoods of many donkey-owning households who use them as a means of sustenance [4]. At the time when this report was written, the licenses of operation of these donkey slaughterhouses had been revoked through a gazette notice No. 50 of April 20, 2020 [29]. This would prevent the theft of donkeys for slaughter and therefore reduce the threat of the diminishing donkey population.

Diseases were also identified as challenges affecting working donkeys in the central highlands. The identified diseases included tetanus, hoof problems which caused lameness, mange as well as endo-parasitic infections. Studies about the prevalence, presentation, and management of tetanus in Kenya were missing, although the number of reported cases was low (personal communication with subcounty veterinary officer Dr. Mulonzi C.N on 20/4/20). Donkeys were naturally susceptible to tetanus due to their normal behavior of rolling on the ground; mostly on soil [30] where tetanus spores could be present, hence predisposing them to tetanus infection. Tetanus was reported to be the most significant disease (Z=5.35) among working donkeys in smallholder farms because its prognosis was guarded and it was mostly fatal for donkeys. The disease can be prevented through vaccination to reduce the chances of infection [31].

Helminthiasis, which was the most common endo-parasitic infection with a reported prevalence of 71.6% [22], reduced the work output of donkeys and consequently the income obtained through them [32]. Helminthiasis was the only disease which was ranked highly among the respondents (Z=3.10) and had a high reported prevalence 71.6%.

Lameness was an indicator a poor welfare status in animals [33] and also affected the work output in donkeys [34]. The prevalence of lameness among working donkeys was 27% in Ethiopia [35]. Similar prevalence studies have not been conducted in Kenya. According to the respondents, diseases had significant impacts based on their effects on work output for the donkeys, reducing productivity, and often causing death. That could explain why the occurrence of wounds was not highlighted as a significant disease in the central highlands of Kenya, although its prevalence among working donkeys was high at 82.3% in Ethiopia [36]. The presence of wounds on donkeys indicated a poor welfare status in animals and predisposed them to tetanus infections [37]. The wounds would often result from friction caused by faulty carts and harnesses as well as using injurious whips and malicious cutting [38]. Whipping was common in Kirinyaga County as a method of directing donkeys on the road in the absence of bits. Malicious cutting was highlighted as a significant challenge affecting working donkeys (Z=2.32). Donkeys were often injured maliciously by the community members when donkeys strayed into their farms and often destroyed their property, and if the resulting conflict was not solved amicably by the warring community members. Malicious cutting often resulted in the death of donkeys because the injuries were too severe to be managed.

Mange was identified among the significant diseases affecting donkeys in the central highland area; because it affected the skin coat appearance of the donkeys, which reduced their market price during the point of sale. Mange also caused separation of donkeys from their owners and discouraged potential clients from hiring the donkeys for use to generate extra income. The respondents also reported that there were risks that the disease was contagious and could be spread to other donkeys in contact with affected donkeys. Although Kyeswa [19] in a study to estimate the prevalence of ectoparasites in Mwingi County, Kenya, did not identify mites in donkeys, the findings from this study show that the disease was ranked highly on significance among respondents.

Addressing these challenges would optimize the benefits of donkeys among smallholder farming systems in Kenya. This would, however, call for a collaborated effort among all stakeholders involved in the value chain of working donkeys.

## Conclusion

The benefits of keeping donkeys in the central highlands of Kenya were for transportation of different kinds of goods as well as for the production of manure. Both of these benefits contributed to income. The income was obtained directly through payment for transport of goods by the donkeys and sale of donkey manure.

Working donkeys were faced by challenges such as rampant theft of donkeys for slaughter, road accidents, malicious injuries, as well as diseases such as tetanus, worms, mange, and hoof problems. These challenges were ranked with significance based on their potential to affect work output, reduce the level of income earned by the households through donkeys or those that caused death.

This study only reports findings based on donkey owner knowledge of the benefits and identification of diseases that affected their donkeys, but future prospective studies should be conducted to determine the animal and herd level prevalence of the identified diseases which were ranked high by respondents.

## Authors’ Contributions

MG designed the study, participated in field data collection, performed the statistical analysis, and wrote the manuscript. JO, RW, and PG participated in the design of the study and reviewed the manuscript. All authors read and approved the manuscript.
